# Cocaine-Induced Acute Extremity Compartment Syndrome: A Rare Case Report

**DOI:** 10.7759/cureus.57297

**Published:** 2024-03-30

**Authors:** Qusai Al-Maharmeh, Ahmad W Haddad, Wassim Abouzeid, Mohammad Kloub, Deema Haddad

**Affiliations:** 1 Internal Medicine, Saint Michael's Medical Center, Newark, USA; 2 Internal medicine, Saint Michael's Medical Center, Newark, USA; 3 Medicine, Jordan University of Science & Technology, Irbid, JOR

**Keywords:** acute leg pain, rhabdomyolysis, fasciectomy, cocaine abuse, acute compartment syndrome of leg

## Abstract

There is a dearth of research connecting acute extremities compartment syndrome to cocaine. Here, we present a case of a forty-year-old guy who is actively using cocaine and comes to the emergency room with excruciating right leg pain and swelling. Physical examination revealed substantial tachycardia, lack of dorsalis pedis pulses, stiff and painful calf muscles, and absence of plantar reflexes in the right lower extremities after sleeping on his right leg. A positive urine drug screen for cocaine, severe rhabdomyolysis, and acute renal damage warranted further laboratory testing. A diagnosis of compartment syndrome was established based on the lack of dorsalis pedis pulses in the right lower extremity and radiographic evidence of oedematous alterations in the calf muscles with perimuscular edema. For this case, acute renal injury was done, and treatment with fluid, hemodialysis, and right lower extremity double-compartment fasciotomies have been used. After that, his clinical situation improved, and no other dialysis sessions were required. Cocaine usage has been linked to rhabdomyolysis; nevertheless, compartment syndrome is an extremely uncommon consequence, particularly in the absence of severe damage or extended immobility.

## Introduction

When intra-compartmental pressures are raised, hypoxia, ischemia, and soft tissue necrosis follow, known as acute compartment syndrome (ACS) [1]. Neurovascular deficiencies, limb loss, and even death are examples of complications [2]. It is essential to diagnose compartment syndrome as soon as possible to avoid the morbidity that results from postponing treatment. The most typical presenting symptom is exaggerated pain [3]. Diagnosing abnormal appearances and underlying causes can be challenging. Infection, rhabdomyolysis, drug usage, surgical placement, intense activity, and more are a few examples [4]. We describe the instance of a guy who, after using one bag of cocaine, slept on his right leg and developed extreme agony. The patient admitted to using cocaine but had not experienced any severe trauma. Following a diagnosis of right leg compartment syndrome, the patient underwent fasciotomies as a form of treatment. We think that using cocaine on its own may contribute to the development of ACS.

This report is essential to the medical community's capacity to react suitably to anomalous presentations and uncommon causal variables. We did not find any case report in the literature for someone who developed compartment syndrome after cocaine with no significant trauma or vigorous exercise.

## Case presentation

A 56-year-old Hispanic male with no known past medical history presented to the emergency department with complaints of sudden-onset right calf pain and swelling, which began approximately 8 hours before presentation. The patient admitted to recent cocaine followed by sleep. Upon awakening, he experienced severe right leg pain and was unable to move the leg or foot. He denied similar symptoms in the past but noted dark urine. He denied any chills, rigors, flank pain, or constipation, any history of trauma, and no previous surgeries. 

Upon examination, the patient's right leg was cold, swollen, hard, and firm to palpation with no sensory or motor dysfunction. Pulse examination using Doppler ultrasound revealed no audible pulses of the dorsalis pedis and posterior tibial arteries. Stryker measurements were obtained, indicating elevated compartment pressures with an anterior compartment pressure of 20 mmHg and a lateral compartment pressure of 88 mmHg, leading to the diagnosis of compartment syndrome.

Complete blood count (CBC) shows a WBC count of 20x10^3^/µL normal range (4.40-11.00x10^3^/uL), hemoglobin level of 18 g/dl normal range (13.5-175 g/dl), suggestive of leukocytosis and hemoconcentration, respectively. A complete metabolic panel (CMP) shows creatinine of 2.6 mg/dl, with a normal range (0.70-1.30 mg/dl), blood urea nitrogen (BUN) of 30 mg/dl, with a normal range (6.0-24.0 mg/dl), and potassium level of 5.2 mmol/L, with a normal range (3.5-5.3 mmol/l), indicating acute kidney injury (AKI). Aspartate aminotransferase (AST) level was 3200 U/L, with a normal range of (10-36 U/L), alanine transaminase (ALT) was 1000 U/L, with a normal range (10-49 U/L), suggestive of acute liver injury, creatine kinase (CK) level of 39000 U/L, with a normal range (52-336 U/L), consistent with a severe muscle injury, likely secondary to compartment syndrome. Urine analysis (UA) shows +3 blood in urine, suggesting myoglobinuria secondary to rhabdomyolysis. Doppler ultrasound of the leg showed no acute or chronic deep vein thrombosis.

**Figure 1:**
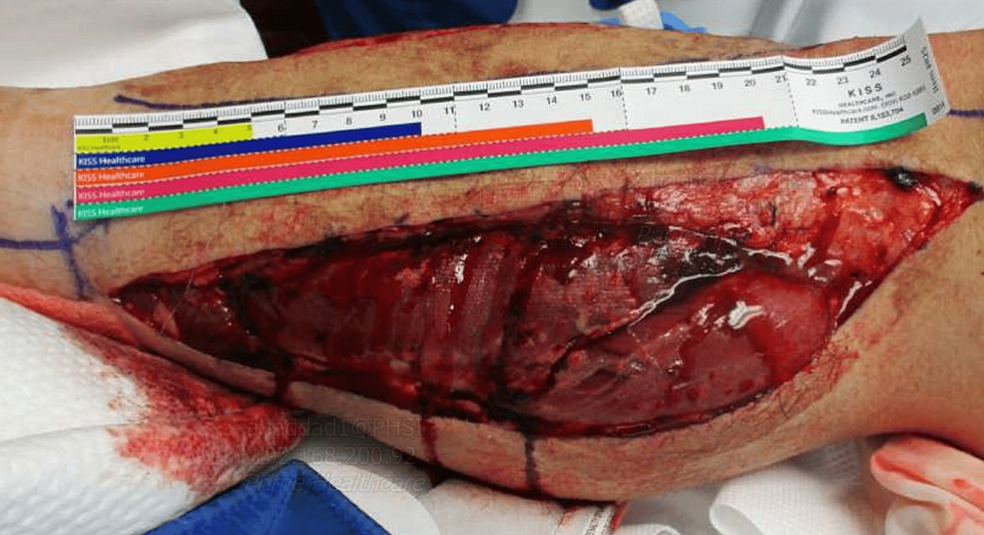
Medial right lower leg fasciectomy This represents the right lower leg after fasciectomy. Muscle swelling was noticed with the clean wound.

**Figure 2:**
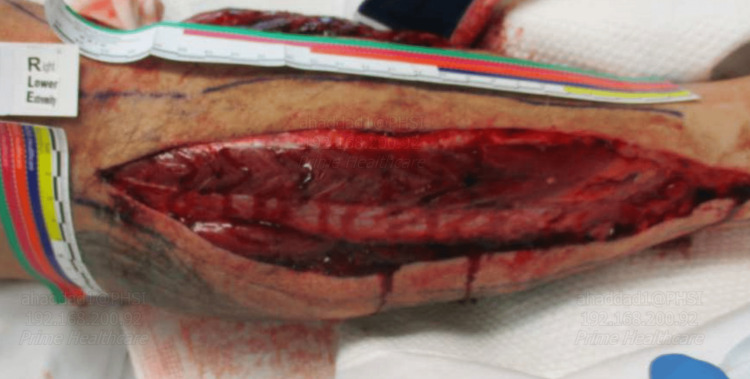
Lateral right lower leg fasciectomy Muscle swelling can also be seen on the lateral side of the right lower leg with the clean wound.

Given the diagnosis of compartment syndrome, the patient was taken for OR a fasciotomy, medially decompressing the superficial as well as the deep posterior compartments. Muscles in these compartments were contractile. The anterolateral incision was then made and carried down through the fascia anterolaterally, with the opening of the fascia on the anterior and lateral compartments after identifying the intramuscular diaphragm and protecting the superficial peroneal nerve. The anterior and lateral compartments appeared contractile, and wet to dry dressings were applied with a plan to vacuum-assisted closure system after 24 hours; the patient was placed in a posterior splint, well padded (Figure 1, 2). Postoperative care closely monitored compartment pressures, vital signs, and neurological status. The pulse immediately returned after the surgery when examined by Doppler. Renal protective measures and electrolyte correction were initiated for acute kidney injury in addition to one hemodialysis session to eliminate the high CK. The patient's creatinine went down to 1.4 mg/dl, with a normal range (0.70-1.30 mg/dl) after two days, with CK going down to 5000 U/L normal range (52-336 U/L). Continuous fluid replacement with ringer lactate at 200 ml/hr for the first 3 days, with pain medications as needed. Substance use disorder counseling and resources were offered to address the underlying cause of the patient's presentation. Follow-up appointments were scheduled for wound care and rehabilitation.

## Discussion

Cocaine is a widely used substance both domestically and internationally. When it comes to drug usage and abuse, it is surpassed only by alcohol. The Drug Abuse Warning Network (DAWN) estimated that drug use-related ER visits accounted for roughly 4.6 million visits in 2009. Of them, about 420,000 visits-or 137.7 visits per 100,000-were linked to cocaine [5]. Cocaine acts as an amphetamine, changing the sympathetic nervous system to cause effects on the heart and blood vessels. It affects the nervous system as well. There are three main sympathomimetic modes of action for cocaine. It also raises the sensitivity of adrenergic nerve endings to norepinephrine, blocks sodium and potassium channels, and inhibits catecholamine reuptake in sympathetic nerve terminals [6].

The toxicity's several causes most likely include severe vasoconstriction that causes ischemia injury as well as direct muscle toxicity. There is more research on the toxicity of cocaine on the heart than on the skeletal muscles. The development of arrhythmias and cardiotoxicity have been linked to many mechanisms, including the blockage of K^+^ channels, elevation of L-type Ca^2+^ channel current, and suppression of Na^+^ influx during depolarization [7]. It is possible to infer a correlation between cardiac and skeletal muscle injury mechanisms. There has been one documented case of cocaine abuse-related compartment syndrome [8].

An increase in interstitial pressure within a closed fascial compartment leading to microvascular impairment is known as compartment syndrome [9]. Both rhabdomyolysis and compartment syndrome might be causes or effects of the condition. Increased capillary basement membrane permeability and inflammatory mediators are the outcomes of ischemic and necrotic muscle. Tissue pressure rises as a result of increased capillary leakage. The reduced blood flow caused by elevated pressure exacerbates ischemia. Acute lower extremity compartment syndrome can be diagnosed clinically. Traditionally, the first sign is discomfort that seems excessive for the exam. Pulse loss is frequently a late symptom. Diagnosis can also benefit from compartment pressure measurements. Fasciotomies of the affected compartments are performed as part of the surgical treatment to release pressure [9]. In rare instances, cocaine has been demonstrated to cause rhabdomyolysis and muscle damage; compartment syndrome brought on by an overdose of either drug has only been documented in one particular case of cocaine usage. Additionally uncommon is compartment syndrome brought on by rhabdomyolysis [10].

Surgical placement, heat damage, infections, rhabdomyolysis, and iatrogenic causes are examples of unusual causes [11]. Increased compartment pressures have been linked to vasoconstrictive substances like cocaine, particularly when paired with high-intensity exercise and creatine supplementation [12].

Compartment syndrome affecting the lower leg and foot typically presents clinically in a manner similar to other affected body regions. In a systematic study, the most sensitive and early clinical indicator of manifest compartment syndrome was found to be pain [13]. The earliest and most common symptom of compartment syndrome is severe, sudden pain. When analgesics are prescribed indiscriminately to individuals experiencing significant pain, it may conceal the primary complaint. Furthermore, drunk individuals have less discomfort, which leads to an underdiagnosis of compartment syndrome [14]. Additionally common in people with compartment syndrome are sensory impairments [13].

Even though diagnostic tools are sold commercially, people who are suspected of having compartment syndrome must undergo a thorough evaluation. However, the surgeon's knowledge of this complication and the appropriate clinical examination are the most crucial steps in detecting compartment syndrome [14]. By applying the piezo-resistance theory to the intracompartmental pressure measurement, invasive measurement provides a rapid and secure means of diagnosing compartment syndrome [14].

Intracompartmental and diastolic blood pressure should be connected, as previously stated. There is still disagreement over the point at which a compartment should be decompressed. Fasciotomy indications nowadays should be based on clinical evidence (neurologic impairments) or a pressure difference of less than 30 mm Hg between the diastolic and compartment pressures [15]. While urgent fasciotomy is unquestionably the best course of action for individuals with compartment syndrome, there are no recommendations in the literature for patients who may be at risk. There are two ways to execute a lower leg fasciotomy: by one incision or through two [14].

In this case, it is highly important to be vigilant about compartment syndrome, especially in substance abuse patients. It is well documented that cocaine with vigorous exercise or creatine supplements, or even trauma, can cause rhabdomyolysis and compartment syndrome. However, this case is unique because the patient does not use any supplements, and no significant trauma or physical stress was appreciated in his case. so cocaine per se can be the trigger of this event.

## Conclusions

This case emphasizes the uncommon but noteworthy correlation between cocaine use and acute extremities compartment syndrome. The diagnosis of compartment syndrome was made based on the cocaine user's presentation of severe leg pain and edema, as well as physical examination and laboratory data. The significance of early detection and treatment in situations of renal injury and limb preservation is highlighted by the prompt intervention of double-compartment fasciotomies and hemodialysis. Even though compartment syndrome is uncommon, doctors should be on the lookout for it when patients with acute limb symptoms use cocaine.
